# Is the state of health of rheumatoid arthritis patients receiving adequate treatment, predictable? - Results of a survey

**DOI:** 10.1186/s12891-015-0567-5

**Published:** 2015-05-06

**Authors:** Rudolf Puchner, Hans Peter Brezinschek, Josef Fritz, Manfred Herold, Monika Mustak, Thomas Nothnagl, Stephan E Puchner, Andrea Studnicka-Benke, Burkhard F Leeb

**Affiliations:** Rheumatologist and Qualified Health Care Manager, Wels, Austria; Rheumatology and Immunology Division, Department of Internal Medicine, Medical University of Graz, Graz, Austria; Department of Medical Statistics, Informatics and Health Economics, Medical University of Innsbruck, Innsbruck, Austria; Department of Internal Medicine I, Medical University of Innsbruck, Innsbruck, Austria; Department of Internal Medicine II, Kaiser Franz Josef Hospital, Vienna, Austria; Department of Medicine II, Centre for Rheumatology, Stockerau State Hospital, Stockerau, Austria; Karl Landsteiner Institute for Clinical Rheumatology, Stockerau, Austria; Department of Orthopaedic Surgery, Medical University of Vienna, Vienna, Austria; Department of Internal Medicine III, Medical University of Salzburg, Salzburg, Austria

**Keywords:** Quality of life and care, Repeated measurements of health levels for individual patients, Intra-class correlation, Level of agreement, Austrian patients with rheumatoid arthritis, The patient’s view

## Abstract

**Background:**

A survey was conducted to evaluate whether a steady improvement in the quality of life of Rheumatoid Arthritis (RA) patients as frequently reported in clinical studies, does actually occur. The focus of this study laid on the personal perception of RA patients. How do patients who have been treated along accepted guidelines see the state of their health and their joint pain at different points in time?

**Methods:**

RA patients were asked to complete a questionnaire and return it to an opinion research centre. The questionnaire, which was developed by the authors, was divided into the areas: demography, symptom description and medical care, as well as the illness in a personal context. Three telephone interviews followed in monthly intervals when the patients´ feelings about their illness, their every-day coping mechanisms and their social lives were rated. Intra-subject correlation and the level of agreement among patients when assessed at three different points within a two month period, was determined.

**Results:**

127 patients replied to the questionnaire. RA exerts a significant impact on a patient’s daily life. Average ratings of current state of health and joint pain (answered on a 5-part scale extending from 1 (very good) to 5 (very bad)) range between 2.6 and 2.9 all three times. However, intra-subject correlation between the different assessment times, is in general quite modest. Concerning the question: “How is your join pain today?” only 14 of 127 participants express identical ratings all three times , while in one third of the participants, a difference of two digits on the 5-part scale, at least twice had to be noticed. Intra-class correlation coefficients between answers at different points are often much smaller than 0.5. Results were similar in all subgroups analysed (men vs. women; patients receiving biologics vs. those not receiving biologics; disease duration ≤3 years vs. 4 to 10 years vs. ≥11 years).

**Conclusion:**

On an individual level personal assessments of health, well-being and joint pain are nevertheless unsteady even within the timeframe of two months. This is why, even now, RA patients still cannot plan their lives as non-affected people can.

**Electronic supplementary material:**

The online version of this article (doi:10.1186/s12891-015-0567-5) contains supplementary material, which is available to authorized users.

## Background

Rheumatoid arthritis (RA) is as a chronic progressive disease and if left untreated, leads to a premature loss of joint function. Irreversible joint damage can occur within one year of first symptoms. Early diagnosis as well as early treatment is therefore of paramount importance [1,2]. There has been a paradigm shift towards an early-adoption of aggressive treatment of RA within the last decades. Invention of effective regimes has improved the therapeutic possibilities dramatically. Whereas until the end of the 1990s a decrease in swollen joints and a reduction of pain was an achievable and acceptable goal, today the overarching principle of all therapeutic efforts is to achieve a state of remission, which can also be defined as a symptom free status [3-5].

Remission is currently defined differently; however, patient reported outcome (PRO) scores are becoming increasingly important [6-18].

The US Food and Drug administration (FDA) defined PROs as follows: “*A patient reported outcome is any report of the status of a patient’s health condition that comes directly from the patient, without interpretation of the patient’s response by a clinician or anyone else”.* Thus, PRO variables are for instance pain, quality of life, medical care, coping mechanisms, subjective health status, physical activity and working ability [15].

Until now, no combined patient reported outcome (PRO) score exists, which is generally accepted for monitoring RA exclusively and which focuses on the perspective of the patient as a primary outcome value [12,14]. Composite indexes, primarily focusing on the opinions of physicians are commonly applied in RA assessment [12,14].

The focus of this study was laid on the personal perception of RA patients. The primary research question of the study was: How do patients with RA who have been treated along accepted guidelines [19,20] see the state of their health, their joint pain and accordingly their well-being at different points in time. Respectively, what is the intra-subject correlation in quality of life assessment when measured over different times?

Is it realistic to postulate a stable, predictable disease status for RA patients, as it is the case in most clinical trials? Alternatively, is the individual perception vastly different from such a situation? Is it true that these patients are not in a position to plan mountaineering or a hiking trip, a journey abroad or a stressful international meeting due to fluctuations in their RA status?

We report here on the results of a survey targeting these very questions.

## Methods

To this end, a questionnaire was developed by the group of authors in a number of meetings together with Karmasin Motivforschung (Motivation Research) – an independent institute that researches communication and target groups. The rheumatologists provided the focus and the specialist input.

After having formulated our research questions a topic guide akin to an interview schedule was developed by the authors. The authors (most of them board members of the Austrian society of rheumatology) intentionally did not use one of the existing tools, as they did not fit our research question, but agreed to create a new questionnaire, based on existing validated generic tools [21]. A Delphi approach including two rounds and a final meeting was applied for consensus-building. Subsequently pilot interviews with volunteer participants (eight patients) were performed, ensuring that all of the interview questions were relevant and appropriate. The interviews were repeated once showing that all of the interview questions were reliable. The pilot and final questionnaires differed only slightly in wording. No patients, who were recruited to take part in the study were interviewed in the pilot phase.

The questionnaire which has a modular approach to disease assessment consists of demographic data, pain variables, pain relieving and intensifying factors, previous treatment procedures, disease related disability, social factors and health related quality of life. Primarily no psychometric testing was performed, as the research should predominantly focus on the course of the disease and not on the personality of patients with rheumatoid arthritis.

Socio-demographic details about sex, age, current place of residence, level of education and disease duration as well as initial clinical data were evaluated with the printed questionnaire before the start of the telephone survey.

Disease severity was defined on 5-part scale (1=very good, 5 =very bad) by the patient. Karmasin Motivforschung was also involved in the development of the questionnaire, namely in the setting and compilation of questions and also gave their support regarding the wording. The printed questionnaires (Additional file 1) ultimately to be filled in personally (paper and pencil), were then distributed to the physicians formerly agreed upon. A nationwide sample was found being more representative for this research question.

These physicians then distributed the appropriate papers to their patients (questionnaire, information hand-out, consent form – to be signed by both the patient and physician) during consulting hours. The physicians explained the study to them and answered any questions. The questionnaires had to be completed by the patients at home and be returned by mail (a stamped, addressed envelope to Karmasin Motivforschung was provided), between the 20^th^ of October 2010 and the 19^th^ of April 2011. This method of procedure was chosen on the one hand, to preclude any influence of the physicians on the patients’ answers and on the other, to preserve their privacy.

All patients fulfilled the 1987 American College of Rheumatology classification criteria [22].

In the subsequent telephone interviews (Additional file 2) performed by employees of Karmasin Motivforschung, the patients´ feelings about their illness, their every-day coping mechanisms and their social lives were rated.

The returned, personally answered questionnaires formed the basis for the three telephone interviews. These questionnaires included the patients´ contact details (including a telephone number that could be used for an interview).

All questionnaires returned before the start of the telephone survey were included as baseline reference data for use in tracking.

Three telephone interviews (CATI Computer Assisted Telephone Interview) lasting roughly fifteen minutes each, followed in the form of a three wave tracking study.

- Baseline call: 21st February to 7th March 2011
- 1st wave of calls: 21st March to 11th April 2011
- 2nd wave of calls: 23rd May to 30th May 2011

The test patients, whose questionnaires had only been returned after the baseline calls had begun, could only be involved from this time onwards. It was only possible for them to be monitored in the 1st and 2nd waves.

Ideally, the test patients were interviewed three times. This was however dependent on their availability and willingness to take part. The structure of the sample was therefore not identical throughout all three component, tracking waves.

Karmasin Motivforschung undertook data collection without any influence exerted by the participating physicians. The project was reviewed and approved by the ethics committee of the Medical University of Graz.

For statistical analysis, telephone interview questions were grouped into the following two categories:

Subjective health status:

The questions assigned to this category were:

- *“How would you describe your current state of health?” (Q1)*
- *“How would you describe your overall feeling of well-being at the present moment?” (Q2)*
- *“How is your joint pain today?” (Q3)*
- *“How happy are you today with the general state of your illness?” (Q4)*

These questions were answered on a scale from one (best) to five (worst).

Quality of life:

The questions assigned to this category were:

- *“To what extent is the quality of your life today influenced by the illness, rheumatoid arthritis?*” (Q5)
- *“Which ramifications does rheumatoid arthritis have for your life today?” (Q6)*
- *“How are you dealing with your illness today?” (Q7)*
- *“Which of the following has a bearing on your quality of life in relation to your illness, today?” (Q8)*
- *“Think of your own personal future in relation to your illness. What attitude do you have to your own future at this time?” (Q9)*

Q5 was answered on a scale from one (not at all) to five (extremely); the other four questions had multiple answers.

Qualitative baseline characteristic data was analysed descriptively, using counts and frequencies.

In addition to descriptive statistics, answers given at different times (baseline, 1st wave and 2nd wave) were compared using the Friedman test for data assessed on an ordinal scale (mostly 1 (e.g. very good) to 5 (e.g. very bad)). Cochran’s Q test was used for the evaluation of data collected from dichotomous answers (i.e. yes/no). Confidence intervals for qualitative data were obtained using the bootstrapping method based on 10,000 replications. Two-sided p-values were calculated with a significance level of α =0.05.

Intra-class (i.e. intra-subject in our study) correlation coefficients (ICCs) for one-way random single measures (type (1, 1)) between different specific times (i.e. baseline vs. 1st wave, baseline vs. 2nd wave and 1st wave vs. 2nd wave) were calculated for ordinal data. For dichotomous data, Cohen’s Kappa was calculated to estimate the level of agreement among the different points in time.

In addition, we performed subgroup analyses for sex (men vs. women), if patients had been receiving biologics or not and for disease duration (≤3 years vs. 4 to 10 years vs. ≥11 years). Single, specific measurements of ordinal data from the groups were compared with Mann–Whitney-U tests/Kruskal-Wallis tests. ICCs were considered significantly different between the two strata if the 95% confidence intervals did not overlap. Statistical analyses were performed using SPSS software (SPSS, Inc., Chicago, Illinois), version 20.

## Results

### Results of the questionnaire

One hundred and twenty-seven patients completed the questionnaire. The sample includes patients from the whole of Austria with a particular emphasis on Lower Austria (57 patients, 45%), Upper Austria (31 patients, 24%) and Tyrol (25 patients, 20%), 14 patients (12%) come from other provinces. These three federal provinces are very similar with respect to their history and their socio-economic status, compared to the other six provinces in Austria, but do display minor topographical differences, the Tyrol being more mountainous.

Level of education: 68 patients (54%) had completed compulsory education, 28 (22%) an education to certificate level and 25 (20%) education to college/university level. 5 (4%) did not answer the question.

Size of community: 80 (63%) of the patients lived in communities of under 5,000 inhabitants; a further 23(18%) in communities of under 50,000 inhabitants; 21(17%) in communities of over 50,000 inhabitants and 3 (2%) did not answer the question.

Eleven percent of the participants are younger than 45, 35% are between 45 and 60 and 53% are 60 and older. The first RA symptoms occurred before the age of 30 in 22% of the patients, in 15% before the age of 40, in a further 22% before the age of 60, and in 10% after the age of 60. Table 1 shows further demographic data and the opinions of patients on their medical care as expressed in their questionnaire answers.

**Table 1:** **Overall patient information and patients’ attitude towards medical care**

**Characteristic**	**Number (% of total)**
Questionnaires completed	127 (100%)
**General patient information**	
Sex	
Male	24 (19%)
Female	103 (81%)
Age	
Younger than 45 years	14 (11%)
45 to 60 years	45 (35%)
Older than 60	67 (53%)
No answer	1 (1%)
First occurrence of RA symptoms	
Before the age of 30	28 (22%)
Between the age of 30 and 40	19 (15%)
Between the age of 40 and 50	28 (22%)
Between the age of 50 and 60	38 (30%)
After the age of 60	13 (10%)
No answer	1 (1%)
Disease duration^1^	
Median (IQR) [years]	8.5 (4–13)
≤3 years	18 (14%)
4 to 10 years	30 (24%)
≥11 years	28 (22%)
No answer	51 (40%)
Severity of RA	
Mild	18 (14%)
Moderate	80 (63%)
Severe	26 (21%)
No answer	3 (2%)
**Medical care**	
Satisfaction with information received from physician during diagnostic process	
Very satisfied	65 (51%)
Satisfied	30 (24%)
Neutral	17 (13%)
Not particularly happy	9 (7%)
Completely dissatisfied	4 (3%)
No answer	2 (2%)
Patient was included in therapeutic decision making by physician	
Yes	105 (83%)
No	16 (13%)
Current medications	
Methotrexate	77 (61%)
Leflunomide	13 (10%)
Sulphasalazine	7 (6%)
Chloroquine/hydrochloroquine	4 (3%)
Concomitant glucocorticoids	56 (44%)
Biologics	36 (28%)
Alternative or complementary remedies	17 (13%)

IQR – interquartile range.

^1^Disease duration could only be calculated if not only the categories, but also the exact age of diagnosis of disease and current age were provided. Since many patients did not provide this information in the questionnaire, disease duration could not be computed for 51 patients.

In 18 patients (14%) rheumatoid arthritis was diagnosed within 3 years before study start, in 30 patients (24%) between 4 and 10 years, and in 28 patients (22%) disease was diagnosed more than 10 years ago. For the remaining patients (40%) exact dates were missing and disease duration could not be calculated.

### Results of the subsequent telephone interviews

A) Subjective health status

The results are summarized in Tables 2 and 3. The answers to all questions were quite similar every time the calls were made and have an average rating of 2.6 to 2.9 with standard deviations ranging from 0.9 to 1.2. Friedman test does not reveal any statistically significant differences between the different times.

**Table 2:** **Summary of answers to the questionnaire at the three different time-points**

	**Baseline (N = 110)**	**1st wave (N = 109)**	**2nd wave (N = 113)**	**p-value**
	**Mean ± SD or pct. (95% CI)**	**Mean ± SD or pct. (95% CI)**	**Mean ± SD or pct. (95% CI)**	
**Category: subjective health status**				
*How would you describe your current state of health?*	2.6 ± 0.9	2.8 ± 1.1	2.8 ± 1.0	0.203^1^
*How would you describe your overall feeling of well-being at the present moment?*	2.7 ± 1.1	2.7 ± 1.2	2.6 ± 1.0	0.606^1^
*How is your joint pain today?*	2.9 ± 1.1	2.8 ± 1.1	2.9 ± 1.1	0.887^1^
*How happy are you today with the general state of your illness?*	2.7 ± 1.2	2.6 ± 1.2	2.8 ± 1.2	0.407^1^
**Category: quality of life**				
*To what extent is the quality of your life today influenced by the illness, rheumatoid arthritis?*	3.2 ± 1.2	2.8 ± 1.2	2.7 ± 1.2	<0.001^1^
*Which ramifications does rheumatoid arthritis have for your life today? (more than one answer possible)*				
The pain stresses me	70.0% (60.9-78.2)	66.1% (56.9-75.2)	67.3% (58.4-76.1)	0.676^2^
The pain affects my daily life	70.0% (61.8-78.2)	49.5% (40.4-58.7)	46.9% (38.1-55.8)	<0.001^2^
The illness has psychological repercussions	51.8% (42.7-60.9)	42.2% (33.0-51.4)	42.5% (33.6-51.3)	0.123^2^
In spite of my illness, full performance is expected of me	26.4% (18.2-34.5)	32.1% (23.9-41.3)	25.7% (17.7-33.6)	0.275^2^
The illness has little or no influence on my life	25.5% (17.3-33.6)	44.0% (34.9-53.2)	36.3% (27.4-45.1)	0.004^2^
The illness has had a negative influence on my social status	22.7% (15.5-30.9)	20.2% (12.8-27.5)	26.5% (18.6-34.5)	0.566^2^
*How do you deal with your illness today? (more than one answer possible)*				
Medication makes handling of the illness easier	90.0% (83.6-95.5)	93.6% (89.0-97.2)	83.2% (76.1-89.4)	0.039^2^
The illness is simply part of everyday life i.e. I have learned to come to terms with it	81.8% (74.5-89.1)	86.2% (79.8-92.7)	83.2% (76.1-89.4)	0.830^2^
I adopted a positive attitude towards the illness	33.6% (25.5-42.7)	63.3% (54.1-72.5)	52.2% (43.4-61.1)	<0.001^2^
The illness is just a burden	17.3% (10.0-24.5)	31.2% (22.9-40.4)	28.3% (20.4-36.3)	0.006^2^
*Which of the following has a bearing on your quality of life in relation to your illness, today? (more than one answer possible)*				
Independence, i.e. doing things that I want to do	82.7% (75.5-89.1)	82.6% (75.2-89.0)	80.5% (72.6-87.6)	0.705^2^
Being active, e.g. sports or going places and doing things	74.5% (66.4-82.7)	76.1% (67.9-83.5)	80.5% (73.5-87.6)	0.809^2^
Social activity, e.g. meeting friends	72.7% (64.5-80.9)	78.9% (71.6-86.2)	83.2% (76.1-89.4)	0.200^2^
Good physical well-being, e.g. being pain-free and mobile	61.8% (52.7-70.9)	82.6% (75.2-89.0)	78.8% (71.7-85.8)	0.001^2^
Other	2.7% (0.0-6.4)	3.7% (0.9-7.3)	3.5% (0.9-7.1)	1.000^2^
*Think of your own personal future in relation to your illness. What attitude do you have to your own future at this time? (single choice)*				
Generally optimistic	54.5% (45.5-63.6)	46.6% (36.9-56.3)	52.4% (42.7-62.1)	0.179^1^
Neutral	40.0% (30.9-49.1)	43.7% (34.0-53.4)	35.0 (26.2-44.7)%	
Generally pessimistic	5.5% (1.8-10.0)	9.7% (3.9-15.5)	12.6% (6.8-19.4)	

N – number of answered questionnaires; SD – standard deviation; CI – confidence interval; percentages (pct.) are based on N; CIs were obtained by bootstrapping method based on 10000 replications; NC – not captured.

^1^p-value of Friedman test; ^2^p-value of Cochran’s Q test.

**Table 3:** **Intra-subject correlation and level of agreement at different times**

	**Baseline vs. 1st wave**	**Baseline vs. 2nd wave**	**1st wave vs. 2nd wave**
**Category: subjective health status**			
*How would you describe your current state of health?*	0.37^1^	0.48^1^	0.47^1^
*How would you describe your overall feeling of well-being at the present moment?*	0.39^1^	0.39^1^	0.40^1^
*How is your joint pain today?*	0.48^1^	0.37^1^	0.40^1^
*How happy are you today with the general state of your illness?*	0.35^1^	0.40^1^	0.46^1^
**Category: quality of life**			
*To what extent is the quality of your life today influenced by the illness, rheumatoid arthritis?*	0.47^1^	0.41^1^	0.56^1^
*Which ramifications does rheumatoid arthritis have for your life today (more than one answer possible)?*			
The pain stresses me	0.45^2^	0.37^2^	0.39^2^
The pain affects my daily life	0.48^2^	0.19^2^	0.29^2^
The illness has psychological repercussions	0.31^2^	0.42^2^	0.33^2^
In spite of my illness, full performance is expected of me	0.41^2^	0.39^2^	0.35^2^
The illness has little or no influence on my life	0.13^2^	0.12^2^	0.37^2^
The illness has had a negative influence on my social status	0.28^2^	0.25^2^	0.26^2^
*How are you dealing with your illness today (more than one answer possible)?*			
Medication makes handling of the illness easier	0.19^2^	0.23^2^	0.08^2^
The illness is simply part of everyday life i.e. I have learned to come to terms with it	0.16^2^	0.36^2^	0.25^2^
I adopted a positive attitude towards the illness	0.23^2^	0.08^2^	0.11^2^
The illness is just a burden	0.32^2^	0.26^2^	0.37^2^
*Which of the following has a bearing on your quality of life in relation to your illness, today (more than one answer possible)?*			
Independence, i.e. doing things that I want to do	0.12^2^	−0.04^2^	0.10^2^
Being active, e.g. sports or going places and doing things	0.11^2^	0.19^2^	0.05^2^
Social activity, e.g. meeting friends	0.14^2^	0.11^2^	0.14^2^
Good physical well-being, e.g. being pain-free and mobile	0.03^2^	−0.04^2^	0.14^2^
Other	0.56^2^	−0.03^2^	−0.04^2^
*Think of your own personal future in relation to your illness. What attitude do you have to your own future at this time? (single choice)*	0.55^1^	0.31^1^	0.53^1^

^1^Intraclass correlation coefficient (ICC) for one-way random single measures; ^2^Cohen’s Kappa.

Only 12%, 10% and 9% of all patients interviewed at baseline, first and second wave respectively, describe their current state of health as very good and only 9%, 13% and 11% of them are free of joint pain when interviewed, all three times.

Intra-subject correlation in the answers between baseline and first wave, baseline and second wave as well as first wave and second wave range between 0.35 and 0.48.

**Figure 1:**
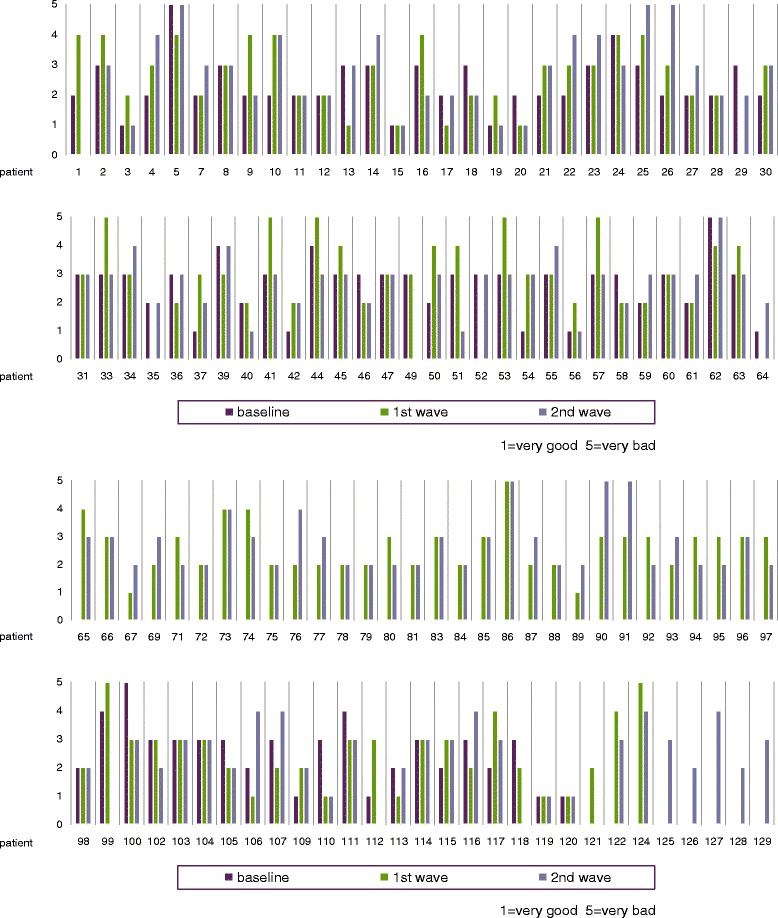
“How would you describe your current state of health?” (Individual ratings).

Overall individual subject ratings for Q1 can be seen in Figure 1 at all three assessment times. Only 20 of 127 participants express identical ratings each time they were monitored. In 29 of the participants, there is a difference of two on the 5-part scale at least two of the times and in four cases, a difference of three also at least twice for over the complete monitoring period.

**Figure 2:**
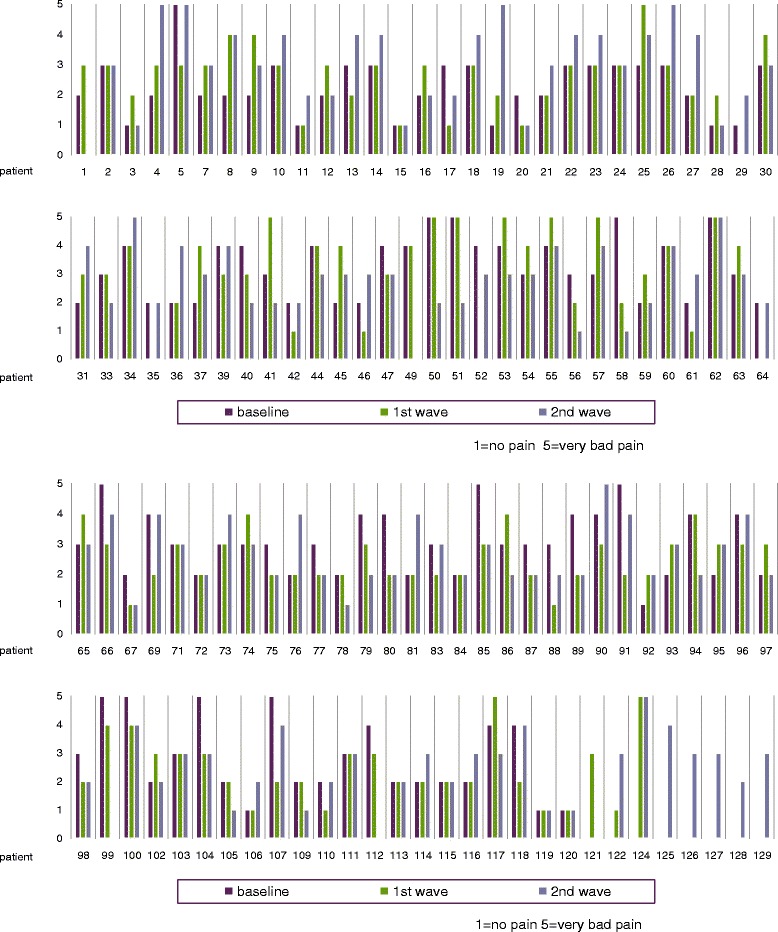
“How is your joint pain today?” (Individual ratings).

An analogue figure for Q3 can be found in Figure 2. Here, only 14 of 127 participants display identical ratings when monitored all three times. In 40 of the participants, there is a difference of two on the 5-part scale, at least twice and in four cases a difference of three also at least twice over the whole monitoring period.

Subgroup analyses were performed for men and women, for patients currently treated with or without biologics and for patients with a disease duration ≤3 years, 4 to 10 years, and ≥11 years. Results are displayed in Table 4. Neither mean values nor ICCs differed significantly between categories in any subgroup. The proportion of patients with identical ratings overall and of those whose ratings differed by at least two, were comparable in all subgroups.

**Table 4:** **Subgroup analyses for sex and treatment with biologics**

	**Baseline**	**1st wave**	**2nd wave**	**ICC baseline vs. 1st wave**	**ICC baseline vs. 2nd wave**	**IC C 1st wave vs. 2nd wave**
	**Mean ± SD**	**Mean ± SD**	**Mean ± SD**			
**Category: subjective health status**						
*How would you describe your current state of health?*						
Men	2.5 ± 0.8	2.6 ± 1.1	2.7 ± 1.1	0.29	0.44	0.56
Women	2.6 ± 0.9	2.8 ± 1.1	2.8 ± 1.0	0.39	0.49	0.44
Biologics as current medication	2.6 ± 0.9	2.7 ± 1.1	2.7 ± 1.1	0.51	0.57	0.54
No biologics as current medication	2.6 ± 0.9	2.9 ± 1.1	2.8 ± 1.0	0.30	0.41	0.43
Disease duration ≤3 years	2.8 ± 0.8	2.8 ± 1.1	2.7 ± 0.8	0.45	0.50	0.50
Disease duration 4 to 10 years	2.7 ± 0.9	2.9 ± 1.2	3.0 ± 1.2	0.57	0.47	0.58
Disease duration ≥11 years	2.4 ± 1.0	2.8 ± 1.1	2.9 ± 0.9	0.41	0.60	0.32
*How would you describe your overall feeling of well-being at the present moment?*						
Men	2.7 ± 1.0	2.6 ± 1.2	2.6 ± 0.9	0.63	0.32	0.48
Women	2.7 ± 1.1	2.7 ± 1.2	2.7 ± 1.1	0.35	0.40	0.39
Biologics as current medication	2.5 ± 1.0	2.6 ± 1.2	2.7 ± 1.2	0.32	0.29	0.38
No biologics as current medication	2.8 ± 1.2	2.7 ± 1.1	2.6 ± 0.9	0.42	0.45	0.42
Disease duration ≤3 years	2.8 ± 1.1	2.5 ± 1.1	2.6 ± 0.9	0.18	0.47	0.46
Disease duration 4 to 10 years	2.8 ± 0.9	2.8 ± 1.2	2.7 ± 1.0	0.57	0.53	0.29
Disease duration ≥11 years	2.6 ± 1.3	2.7 ± 1.1	2.8 ± 1.1	0.59	0.34	0.28
*How is your joint pain today?*						
Men	3.0 ± 1.0	2.7 ± 1.0	2.9 ± 1.2	0.28	0.20	0.25
Women	2.9 ± 1.2	2.8 ± 1.1	2.8 ± 1.1	0.52	0.42	0.42
Biologics as current medication	2.7 ± 1.0	2.8 ± 1.2	2.9 ± 1.2	0.69	0.44	0.57
No biologics as current medication	3.0 ± 1.2	2.8 ± 1.1	2.9 ± 1.1	0.39	0.34	0.31
Disease duration ≤3 years	2.9 ± 1.2	2.6 ± 1.3	2.8 ± 1.2	0.25	0.23	0.64
Disease duration 4 to 10 years	2.7 ± 1.0	3.0 ± 1.2	3.0 ± 1.1	0.60	0.50	0.45
Disease duration ≥11 years	2.9 ± 1.3	2.7 ± 1.2	3.0 ± 1.0	0.48	0.20	0.22
*How happy are you today with the general state of your illness?*						
Men	2.7 ± 1.2	2.6 ± 1.3	2.7 ± 1.4	0.24	0.46	0.65
Women	2.7 ± 1.2	2.7 ± 1.1	2.8 ± 1.2	0.38	0.38	0.41
Biologics as current medication	2.4 ± 1.0	2.7 ± 1.2	2.7 ± 1.3	0.52	0.41	0.48
No biologics as current medication	2.8 ± 1.3	2.6 ± 1.2	2.8 ± 1.2	0.28	0.39	0.46
Disease duration ≤3 years	2.6 ± 1.4	2.5 ± 1.2	2.5 ± 1.3	0.46	0.16	0.39
Disease duration 4 to 10 years	2.5 ± 1.0	2.9 ± 1.3	2.8 ± 1.2	0.47	0.47	0.73
Disease duration ≥11 years	2.9 ± 1.4	2.6 ± 1.1	2.9 ± 1.3	0.25	0.51	0.24
**Category: quality of life**						
*To what extent is the quality of your life today influenced by the illness, rheumatoid arthritis?*						
Men	3.0 ± 1.3	2.8 ± 1.5	2.6 ± 1.2	0.33	0.39	0.58
Women	3.2 ± 1.2	2.8 ± 1.2	2.6 ± 1.2	0.51	0.42	0.56
Biologics as current medication	3.1 ± 1.2	2.6 ± 1.2	2.7 ± 1.3	0.39	0.37	0.64
No biologics as current medication	3.2 ± 1.2	2.8 ± 1.3	2.7 ± 1.1	0.51	0.44	0.52
Disease duration ≤3 years	3.1 ± 1.1	2.3 ± 1.1	2.5 ± 1.0	0.03	0.07	0.21
Disease duration 4 to 10 years	3.1 ± 1.0	3.0 ± 1.3	2.8 ± 1.3	0.52	0.49	0.56
Disease duration ≥11 years	3.2 ± 1.3	2.6 ± 1.2	2.7 ± 1.2	0.48	0.53	0.62

SD – standard deviation; ICC - Intraclass correlation coefficient for one-way random single measures.

No statistically significant differences between men and women, between use of biologics and no biologics, and between disease duration subgroups were observed.

B) Quality of life

The results are summarized in Tables 2 and 3. For Q5 and some elements of Q6, Q7 and Q8 statistically significant (p-value < 0.05), differences in answers at different times could be detected.

The illness encroaches upon the quality of life, average rating answers to Q5 being 3.2 ± 1.2, 2.8 ± 1.2 and 2.7 ± 1.2 at the three different times. The answers of only 12 of 129 participants (9.3%) were the same all three times. In 42 of the participants (32.6%), there was a difference of two on the 5-part scale at least twice and in ten cases (7.8%) a difference of three at least twice over the entire monitoring period. Intra-subject correlations between single times range from between 0.41 to 0.56.

The main implications for RA patients as evaluated with Q6 are stress due to pain, and interference with their daily lives, which is of relevance for more than half of the patients.

In dealing with the illness (Q7), medication and the integration of the illness into day-to-day life are most important: 82% to 94% of all patients handle the disease this way.

No levels of agreement on single elements of Q6 and Q7 reaching a value greater than 0.5, but there is some low level agreement. For example the elements “The illness has little or no influence on my life”, ”Medication makes handling of the illness easier” and “I adopted a positive attitude towards the illness” reached levels of agreements of under 0.15.

Only a small proportion of all patients (5%, 9% and 12% at each interview wave, respectively) are generally pessimistic regarding their own future (Q9), while 55%, 47% and 51% are generally optimistic. The rest has a neutral view. Intra-subject correlation between the different times range from 0.31 to 0.55 for this question.

Subgroup analysis of Q5 for men and women and for patients receiving or not receiving biologics showed similar results on all levels with no relevant deviation (Table 4). For the subgroup with a disease duration ≤3 years ICCs were considerably smaller than in patients with longer disease duration, but results were not statistically significant.

## Discussion

The results derived from the tracking system demonstrate those issues generally seen as cornerstones of the clinical picture, independently of the selected times of monitoring. We were concerned with highlighting those dimensions of the illness that occur with regularity and defining patients´ handling of their disease.

The participating patients can be regarded as an average RA population, expressing acceptable mean ratings for satisfaction with disease status, pain, quality of life and general health.

The disease has a bearing on the quality of life, which each individual patient experiences differently. Over time, patients necessarily come to terms with this situation, which in turn lessens the relevance of the health impairment.

The illness has a significant influence on the life of an RA patient, in particular the symptom of pain. The results are very much in line with a study by Hewlett et al. [23].

In 2011 a EULAR initiative devised a Rheumatoid Arthritis Impact of Disease (RAID) score; a patient derived composite measure of the impact of RA. Parameters of major importance for the patients were pain, followed by physical limitations, fatigue, emotional well-being, and sleep as well as coping mechanisms [24-26]. Again, our results support these findings.

One fundamental conclusion from our study, and a strength of the research, is that the individual patient’s self-appraisal of their disease is quite mutable. Although means and percentages show markedly uniform patterns and acceptable results for the observation period, intra-subject correlation and level of agreement at different times, disappointingly is quite modest in general: High correlation (ICC/kappa ≥ 0.5) was hardly detected in any answers. Most correlation was only moderate (0.25 – 0.5) or even weak (0–0.25). The pattern was similar in all subgroups analysed.

These conclusions are valid for all questions in the two categories (subjective health status and quality of life).

Specifically, the answers to the question on the current state of health of participants are consistent with this observation. The means calculated of the three waves of telephone interviews are quite similar and at 2.6, 2.8 and 2.8 and show no statistically significant differences. Nevertheless, intra-subject correlations only range from 0.38 to 0.50 – which is only a modest relationship but still higher than for many other questions. A very similar picture could be seen for joint pain. The number of participants whose answers differed by at least two at different times, was surprisingly high.

We only can speculate about the factors causing the significant differences between the three waves detected for the answers to the question to what extent the participant’s quality of life is influenced by the illness at that moment, as well as some sub-elements of the multiple choice questions. In a study population with a majoritarian long disease duration such differences were not expected. Aside from the fact that disease activity fluctuations could give the reason for such differences, a growing indifference to the questionnaire and impatience with the telephone calls could be - at least in part - responsible for this phenomenon. Although no differences registered at different times were observed for most of the questions, this is a point, which remains open and is worth further exploration in the future.

Another shortcoming of the study lies in the lack of application of a widely used patient reported outcome measure used in RA. For example, the Routine Assessment of Patient Index Data 3 (RAPID3) [16], the self-administered, Rheumatoid Arthritis Disease Activity Index-5 (RADAI-5) [27], the Health Assessment Questionnaire (HAQ) [28] or the Rheumatoid Arthritis Quality of Life (RAQoL) questionnaire [29], were not captured, but this was to ensure to exert no influence upon our patients. We also did not calculate disease activity like Disease Activity Score 28 (DAS 28) [30] or Clinical disease activity index (CDAI) [31], as a decision was reached not to carry out routine consultations with a rheumatologist during the two month observation time, in order not to influence the telephone Interviews. Therefore, we were not able to compare our newly developed questionnaire with well-established scores or measurements and could not perform a validation procedure. No validation data of the questionnaire in RA is a limitation. We adapted our questionnaire to a telephone survey in the form of three-wave tracking and mostly used numerical rating scales for items like pain, physical function and global health assessment. We found that none of the standardised questionnaires would have been suited to the research question.

Individual assessment of health levels, well-being, joint pain and the quality of life is quite unsteady, even within a time frame of about two months. Due to disease fluctuation, a prediction of health levels, personal well-being and joint pain as well as the quality of life for the individual patient is still in the need of improvement. This of course has major consequences for the individual patient; even nowadays, the planning of activities for the nearest future can create difficulties for them and their environment, irrespective from modern diagnostic techniques as well as from the availability of adequate therapy. Far more individualised treatment schedules adapted to the needs of each single patient are mandatory. This obviously also implies high patient involvement into therapeutic decision-making.

## Conclusions

There is consensus amongst RA patients on some overall key statements with respect to the course of the disease. On an individual level, however, most patients do not achieve a truly steady state of disease even within the short time-frame of two months and therefore they are limited in planning their near future. The individual course of disease in RA is not a simple linear linking of two observations, as clinical trial charts often give cause to believe. High fluctuation between two observations is the rule rather than the exception.

## Additional files

Additional file 1:
**Questionnaire 1 Puchner-Leeb Study-260415.**
Additional file 2:
**Questionnaire 2 Puchner-Leeb Study-260415.**

## Abbreviations

CATI: Computer assisted telephone interview
CDAI: Clinical disease activity index
DAS28: Disease activity score in 28 joints
EULAR: European League Against Rheumatism
FDA: Food and Drug Administration
HAQ: Health Assessment Questionnaire
ICC: Intra-class correlation coefficient
PRO: Patient related outcome
Q: Question
RA: Rheumatoid arthritis
RADAI-5: Rheumatoid arthritis disease activity index-5
RAID: Rheumatoid arthritis impact of disease
RAPID3: Routine assessment of patient index data 3
RAQuOL: Rheumatoid arthritis quality of life questionnaire
