# Supplementary material for: Is the state of health of rheumatoid arthritis patients receiving adequate treatment, predictable? - Results of a survey
Source: BMC Musculoskelet Disord. 2015 May 6;16:109. doi: 10.1186/s12891-015-0567-5 (PMC4427952; doi:10.1186/s12891-015-0567-5)
Supplement: Additional file 1: — Questionnaire 1 Puchner-Leeb Study-260415. [file 12891_2015_567_MOESM1_ESM.pdf]

Karmasin Motivforschung Ges.m.b.H.
Anastasius Grün-Gasse 32, 1180 Wien
Tel.: 01/470 47 24-0

Karmasin
Motivforschung

Welcome to the questionnaire on the demographics of rheumatoid arthritis

1. How old were you when the first symptoms of rheumatoid arthritis occurred? _______________ years

2. How old were you when the diagnosis rheumatoid arthritis was made?

   _______________ years

3. What were your first symptoms of rheumatoid arthritis?
   (more than one answer possible)

   ☐ swollen, painful joints
   ☐ foot/knee / ankle pain
   ☐ painful handshake
   ☐ extreme fatigue
   ☐ finger/wrist pain
   ☐ lower back/shoulder pain
   ☐ morning stiffness
   ☐ other - namely: ___________________

4. How long did it take for a diagnosis of rheumatoid arthritis to be made after first symptoms?
   (one answer)

   - up to 3 months
   - 4 to 6 months
   - 7 to 12 months
   - 13 to 24 months
   - 3 to 4 years
   - 5 years and over

5. Which kind of doctor did you initially consult after first symptoms?
   (one answer)

   - general practitioner
   - specialist for internal medicine
   - specialist for orthopaedics
   - rheumatologist in private practice
   - rheumatologist in a hospital
   - other - namely: ________________________

6. How many doctors did you have to consult before the diagnosis of rheumatoid arthritis was made?
   (one answer)

   - 1 doctor
   - 2-3 doctors
   - 4-5 doctors
   - more than 5 doctors

7. Which kind of doctor diagnosed rheumatoid arthritis?
(one answer)

- general practitioner
- specialist for internal medicine
- specialist for orthopaedics
- rheumatologist in private practice
- rheumatologist in a hospital
- other - namely: ______________________

8. How happy were you with the information supplied to you by the doctor who diagnosed rheumatoid arthritis?

On a scale of 1 to 5:
1 signifying very happy and
5 not at all happy
Fractional grading is also possible!

very happy	1 ☐	2 ☐	3 ☐	4 ☐	5 ☐	not at all happy

9. Were you consulted by your doctor on the choice of therapy?

- yes
- no

10. Which of the following aspects regarding medical care are of importance to you?

On a scale of 1 to 5:
1 signifying very important and
5 not at all important
Fractional grading is possible!

	**very important**				**not at all important**
sufficient patient / doctor consultation	1 ☐	2 ☐	3 ☐	4 ☐	5 ☐
good explanation of the illness	1 ☐	2 ☐	3 ☐	4 ☐	5 ☐
sufficient sensitivity shown by physicians	1 ☐	2 ☐	3 ☐	4 ☐	5 ☐
sufficient care taken when prescribing medication	1 ☐	2 ☐	3 ☐	4 ☐	5 ☐
a right for the patient to have a say in the choice of therapy	1 ☐	2 ☐	3 ☐	4 ☐	5 ☐
swiftly scheduled appointments	1 ☐	2 ☐	3 ☐	4 ☐	5 ☐

11. Which medication are you taking presently?
(more than one answer possible)

- Ebetrexat®, Methotrexat Ebewe® (Methotrexate)
- Salazopyrin® (Sulfasalazine)
- Arava® (Leflunomide)
- Resochin® (Chloroquine)
- Quensyl® (Hydroxychloroquine)
- Sandimmun® (Ciclosporine, Cyclosporine A)
- Ridaura® (Gold as tablets/oral Gold)
- Tauredon® (Gold as an injection/parenteral Gold)
- Aprednislon®, Urbason® (Cortisone)
- Enbrel® (Etanercept)
- MabThera® (Rituximab)
- Remicade® (Infliximab)
- Orencia® (Abatacept)
- Humira® (Adalimumab)
- RoActemra® (Tocilizumab)

- other - namely: ____________________

12. Do you also make use of complementary or alternative medicine?

- yes
- no

If yes, which? ____________________

13. Which form of medication do you prefer?
(One answer)

- tablets
- self-injection
- infusion

} → **continue to question 14**

- no preference → **continue to question 15**

14. What are your reasons for your preference for **tablets or injections or infusions**?

15. Which sources of information have you used in order to learn about rheumatoid arthritis? **(please tick where applicable!)**

How important do you think these sources of information were? **(Please assess every source!)**

On a scale of 1 to 5:
1 signifying very important and

5 not at all important
Fractional grading is possible

How important do you think this source is?

	**I have used this source**	**very important**				**not at all**
reference journals/ books	☐	1 ☐	2 ☐	3 ☐	4 ☐	5 ☐
newspaper articles or TV/radio broadcasts	☐	1 ☐	2 ☐	3 ☐	4 ☐	5 ☐
information leaflet with medicines	☐	1 ☐	2 ☐	3 ☐	4 ☐	5 ☐
internet	☐	1 ☐	2 ☐	3 ☐	4 ☐	5 ☐
homepage / product webpage of manufacturer	☐	1 ☐	2 ☐	3 ☐	4 ☐	5 ☐
information from pharmaceutical companies	☐	1 ☐	2 ☐	3 ☐	4 ☐	5 ☐
lectures and information evenings on rheumatism (e.g. Mini Med in Austria)	☐	1 ☐	2 ☐	3 ☐	4 ☐	5 ☐
patient self-help groups	☐	1 ☐	2 ☐	3 ☐	4 ☐	5 ☐
discussion with fellow patients	☐	1 ☐	2 ☐	3 ☐	4 ☐	5 ☐
your doctor	☐	1 ☐	2 ☐	3 ☐	4 ☐	5 ☐
your chemist	☐	1 ☐	2 ☐	3 ☐	4 ☐	5 ☐
family	☐	1 ☐	2 ☐	3 ☐	4 ☐	5 ☐
friends	☐	1 ☐	2 ☐	3 ☐	4 ☐	5 ☐
other – namely:	☐	1 ☐	2 ☐	3 ☐	4 ☐	5 ☐

16. Were you in employment prior to your illness?

- yes → **continue to question 17**
- no → **continue to question 19**

17. Did you have to give up your job as a result of the illness?

- yes → **continue to question 18**
- no → **continue to question 19**

18. How many years after the start of your illness did you have to give up your job?

- up to 1 year after the start

- 2 to 5 years after the start
- 6 to 10 years after the start
- over 10 years after the start

19. In your present situation, how important is financial help in the following areas?

On a scale of 1 to 5:
1 signifying very important and
5 not at all important
Fractional grading is possible

	very important				not at all important
medication	1 ☐	2 ☐	3 ☐	4 ☐	5 ☐
mobility and other aids	1 ☐	2 ☐	3 ☐	4 ☐	5 ☐
health spa rehabilitation treatment	1 ☐	2 ☐	3 ☐	4 ☐	5 ☐
rehabilitation (e.g. physiotherapy)	1 ☐	2 ☐	3 ☐	4 ☐	5 ☐
help in the home	1 ☐	2 ☐	3 ☐	4 ☐	5 ☐
care (i.e. help with personal hygiene)	1 ☐	2 ☐	3 ☐	4 ☐	5 ☐
cost of transport to doctors, therapists or to hospital	1 ☐	2 ☐	3 ☐	4 ☐	5 ☐

20. How did your family and friends react to your diagnosis of rheumatoid arthritis **(more than one answer possible)**

- with concern
- with sympathy
- with a lack of understanding
- with offers of help
- my illness was not taken seriously
- with withdrawal
- nothing changed, everything was as before the illness
- little or nothing was known about my illness
- other - namely: ________________________

21. How do you estimate the state of your health will be in 10 years? **(one answer)**

- unchanged
- better
- worse

A FEW MORE QUESTIONS ON STATISTICS

Sex:

- female
- male

Age: _______________ (please fill in exact age and also tick below!)

- up to 30 years
- 31 to 45 years
- 46 to 60 years
- over 60 years

Level of severity of the illness rheumatoid arthritis:

- mild
- moderate / middling
- severe

Marital status:

- single
- married / cohabiting
- divorced / separated
- widowed

Level of education reached in the Austrian school system:

- nine compulsory years of school
- compulsory schooling plus apprenticeship
- vocational school / commercial school
- secondary school without a certificate
- secondary school with leaving certificate
- university

Occupation: which career category did / do you belong in?

- professional
- owner/director of a large company
- owner of a smaller company
- employee with minimal qualifications
- civil servant
- employee with superior qualifications
- civil servant with executive powers
- blue collar worker
- skilled worker (incl. apprentices)
- farmer
- in training
- home maker / on maternity leave
- retired

Total monthly income of all persons living in your household together. Please estimate if you do not have an exact sum.

- up to € 1.500,--
- up to € 2.100,--
- up to € 3.000,--
- up to € 3.000,--

Province: Which Austrian province do you live in?

- Vienna
- Lower Austria
- Burgenland
- Styria
- Carinthia
- Upper Austria
- Salzburg
- Tyrol
- Vorarlberg

What is the population of your town or village?

- up to 5.000
- up to 50.000
- up to 50.000

What is the distance between your town or village and the provincial capital?

- under 20 km
- 21 to 50 km
- 51 to 100 km
- over 100 km

Which type of doctor is currently treating your rheumatoid arthritis?
(one answer)

- general practitioner
- specialist for internal medicine
- specialist for orthopaedics
- rheumatologist in private practice
- rheumatologist in a hospital
- other - namely: ________________________

Which means of transport do you usually use when seeing the doctor that treats your rheumatoid arthritis?
(more than one answer possible)

- underground
- tram
- bus
- train
- car
- on foot
- friends or relatives take me
- other - namely: _______________

How long does your trip to see the doctor that treats your rheumatoid arthritis take?
(one answer)

- up to15 minutes
- up to 30 minutes
- up to 1 hour
- up to 2 hours
- over 2 hours

Karmasin Motivforschung Ges.m.b.H.
Anastasius Grün-Gasse 32, 1180 Wien
Tel.: 01/470 47 24-0

Karmasin
Motivforschung

Please inform us of the date on which you completed this questionnaire:

__________ __________ __________
day month year

Please inform us of your contact details for the telephone survey:

Name: ____

Telephone number: ____

Email Address (optional): ____

Postal Address (optional):
____

Participant number: 1
(consecutively numbered)

Roche

Die Umsetzung des Projektes erfolgt mit freundlicher Unterstützung von Roche Austria.
Die Datenerhebung wird von Karmasin.Motivforschung Ges.m.b.H. durchgeführt.
Version 1.0, 5.10.2009
