# Supplementary material for: Is the state of health of rheumatoid arthritis patients receiving adequate treatment, predictable? - Results of a survey
Source: BMC Musculoskelet Disord. 2015 May 6;16:109. doi: 10.1186/s12891-015-0567-5 (PMC4427952; doi:10.1186/s12891-015-0567-5)
Supplement: Additional file 2: — Questionnaire 2 Puchner-Leeb Study-260415. [file 12891_2015_567_MOESM2_ESM.pdf]

Karmasin Motivforschung Ges.m.b.H.
Anastasius Grün-Gasse 32, 1180 Wien
Tel.: 01/470 47 24-0

Questionnaire for a telephone survey in three waves (base-line call, 1st wave after 1 month, 2nd wave after 3 months)

Hello, my name is……… and I am from the Karmasin independent market research institute. Not so long ago you received a questionnaire in the post and agreed to take part in a survey on the subject of rheumatoid arthritis (chronic polyarthritis). Would you have time for a few questions? The interview will take about 10-15 minutes.

1. How would you describe your current state of health?

On a scale of 1 to 5
1 signifying very good
5 signifying very bad
Fractional grading is possible!

Very good	1	2	3	4	5	Very bad

2. And how would you describe your own personal well-being at the present moment?

On a scale of 1 to 5
1 signifying very good
5 signifying not at all good
Fractional grading is possible!

I am feeling great today	1	2	3	4	5	I am not feeling at all good today

3. How is your joint pain today?

On a scale of 1 to 5
1 signifying no pain
5 signifying very bad pain
Fractional grading is possible!

No pain	1	2	3	4	5	Very bad pain

4. How happy are you today with the general state of your illness?

On a scale of 1 to 5
1 signifying happy
5 signifying not at all happy
Fractional grading is possible!

Very happy	1	2	3	4	5	Not all happy

5. To what extent is the quality of your life today influenced by the illness rheumatoid arthritis?

On a scale of 1 to 5
1 signifying that the illness does not affect my quality of life today at all
5 signifying that the illness affects my quality of life badly, today
Fractional grading is possible!

The illness does not affect my quality of life today at all	1	2	3	4	5	The illness affects my quality of life today, badly,

6. What ramifications does the illness rheumatoid arthritis have for your life today?
(multiple answers possible)

- ☐ the illness affects my day to day life greatly e.g. getting dressed, personal hygiene, eating and drinking, coping with the housework etc.
- ☐ the illness affects my mental well-being
- ☐ the pain stresses me
- ☐ everyone around me expects maximum performance in spite of the illness
- ☐ the illness has had a negative influence on my social status
- ☐ the illness has only had a minor influence on my life

7. How do you deal with your illness today?
(multiple answers possible)

- ☐ the illness is just a part of my everyday life i.e. I have learned to come to terms with it
- ☐ the illness is just a burden
- ☐ medication makes handling of the illness easier
- ☐ I have developed a positive attitude to the illness

8. Which of the following has a bearing on your quality of life in relation to your illness, today?
(multiple answers possible)

- being active e.g. doing sports, going places, doing things

- independence i.e. doing what I want to do
- physical well-being e.g. being pain-free and mobile
- social activities such as meeting friends
- other - namely:_______________________

9. Thinking of your own personal future in relation with your illness: what attitude do you have to your own future at this time
(1 answer)

- generally optimistic
- neutral
- generally pessimistic

10. In your opinion, how do the following factors influence your illness at the present time?

On a scale of 1 to 5
1 signifying a positive influence
5 signifying a negative influence
Fractional grading is possible!

	**has a positive influence on my illness**				**has a negative influence on my illness**
personal well-being	1	2	3	4	5
weather	1	2	3	4	5
heat	1	2	3	4	5
cold	1	2	3	4	5
stress /excitement	1	2	3	4	5
worries	1	2	3	4	5
physical strain / exertion	1	2	3	4	5
personal surroundings (e.g. friends / family)	1	2	3	4	5
physical activity / sports	1	2	3	4	5
communication with other RA/cP patients	1	2	3	4	5
prescribed drugs	1	2	3	4	5
recent consultation with a doctor	1	2	3	4	5

11. How does your illness affect your social life at the moment?
(1 answer)

☐ I avoid having a social life e.g. meeting friends, going to the cinema or the theatre etc.
☐ my social life is unchanged in spite of the illness
☐ I have more of a social life due to the illness

12. Do you participate in a patient self-help group at the moment?

- yes → **continue to question 13**
- no → **continue to question 14**

13. **If you do participate in a patient self-help group**: What are your reasons for participating in a patient self-help group?

14. **If you do not participate in a patient self-help group**: What are your reasons for not participating in a patient self-help group?

15. What medication do you currently take?
(more than one answer possible)

- Ebetrexat®, Methotrexat Ebewe® (Methotrexate)
- Salazopyrin® (Sulfasalazine)
- Arava® (Leflunomide)
- Resochin® (Chloroquine)
- Quensyl® (Hydroxychloroquine)
- Sandimmun® (Ciclosporin, Cyclosporin A)
- Ridaura® (Gold in tablet form/oral Gold)
- Tauredon® (Gold in as an injection/parenteral Gold)
- Aprednislon®, Urbason® (Cortisone)
- Enbrel® (Etanercept)
- MabThera® (Rituximab)
- Remicade® (Infliximab)
- Orencia® (Abatacept)
- Humira® (Adalimumab)
- RoActemra® (Tocilizumab)

- other - namely: ______________________________

16. Are you also making use of complementary or alternative medicine?

- yes
- no

If yes, which? ______________________________

17. Do you have a say in the choices regarding your therapy at the moment?

- yes
- no

18. How important are the following factors with regard to the treatment of your illness at the present time?

On a scale of 1 to 5
1 signifying very important
5 signifying not at all important
Fractional grading possible!

	**very importan t**				**not at all importan t**
remission i.e. being free of symptoms	1	2	3	4	5
being free of pain	1	2	3	4	5
being healed	1	2	3	4	5
no restrictions on mobility	1	2	3	4	5
everything should stay as it is	1	2	3	4	5
effective medication with fewer side effects	1	2	3	4	5
minimal effort e.g. in relation to the number of medical consultations and travel there	1	2	3	4	5

19. What influence does the illness have today on your social surroundings, your family and friends? **(more than one answer possible)**

- my family shows great sympathy and support
- my family is under psychological stress due to my illness
- my family cannot accept my illness
- my family is withdrawing from me
- my family is uninfluenced by my illness

→ this question will not be asked in the next survey after one month

20. How important are the following ways of supporting you in dealing with your illness at the present time?

On a scale of 1 to 5
1 signifying very important
5 signifying not at all important
Fractional grading is possible!

	**very importan t**				**not at all importan t**
help with your work e.g. keeping house, with gardening, going shopping	1	2	3	4	5

emotional support e.g. being encouraging either face to face or on the phone, sensitivity, a shoulder to lean on	1	2	3	4	5
financial help from the state e.g. disability benefits, medical exemption from prescription fees, financing of medication / health spa treatments	1	2	3	4	5

21. Do you have financial problems due to your illness at the present time?
(more than one answer possible)

- yes I do, due to:
- the cost of medication not paid for by the health service
- the cost of mobility aids not paid for by the health service
- travel costs for visits to the doctor
- the cost of help in the home
- the cost of in-patient rehabilitation
- the cost of therapy
- losing my job
- other - namely: ____
- no

22. Which of the following situations stress you, due to the illness, at the present time?
(more than one answer possible)

- **pronounced side effects of medication**
- long distances to your doctor
- high financial commitment for medication, mobility aids, transport etc.
- **in-patient care in hospital**
- **complex administration of medication**
- **your physical limitations**
- **the need to ask for help from family and friends**
- other - namely: ____

Karmasin Motivforschung Ges.m.b.H.
Anastasius Grün-Gasse 32, 1180 Wien
Tel.: 01/470 47 24-0

Karmasin
Motivforschung

→ items in bold type will be asked three times

23. If you were able to direct some requests regarding your illness at those in power in the health service, what would those requests be?
